# Genome skimming provides evidence to accept two new genera (Apiaceae) separated from the *Peucedanum* s.l.

**DOI:** 10.3389/fpls.2024.1518418

**Published:** 2025-01-20

**Authors:** Bo-Ni Song, Chang-Kun Liu, Jiao-Jiao Deng, Wei-Yan Tan, Song-Dong Zhou, Xing-Jin He

**Affiliations:** ^1^ Key Laboratory of Bio-Resources and Eco-Environment of Ministry of Education, College of Life Sciences, Sichuan University, Chengdu, China; ^2^ College of Resources Environment and Chemistry, Chuxiong Normal University, Chuxiong, China

**Keywords:** phylogenomics, divergence time, plastome, *Shanopeucedanum*, *Sinopeucedanum*

## Abstract

**Background:**

The *Peucedanum* s.l. genus, the backbone member of subfamily Apioideae, includes many medically and economically important plants. Although previous studies have proved that the genus was not a natural taxonomic unit and taxonomists also conducted several taxonomic revisions for taxa of this genus, classifications of numerous taxa of the genus still have not been satisfactorily resolved, especially for those endemic to China. Therefore, we conducted a comprehensive taxonomic revision of taxa within the polyphyletic *Peucedanum* s.l. genus in this study.

**Methods:**

We used two molecular datasets (103 plastomes and 43 nrDNA sequences) generated by genome skimming to reconstructed a reliable phylogenetic framework with high support and resolution. In addition, we also investigated the divergence time of core clade of endemic taxa.

**Results and Discussion:**

Both analyses failed to recover *Peucedanum* s.l. as a monophyletic group and robustly supported that *P. morisonii*, the representative of *Peucedanum* s.s., was distantly related to other *Peucedanum* s.l. members, which implied that these *Peucedanum* s.l. taxa were not “truly *Peucedanum* plants”. Among these *Peucedanum* s.l. members, plastid-based phylogenies recognized two monophyletic clades, clade A (four species) and clade B (10 taxa). Meanwhile, obvious recognized features for morphology, plastome, and chromosome number for each clade were detected: dorsally compressed and glabrous mericarps with filiform dorsal ribs, winged lateral ribs, numerous vittae in commissure and each furrow, IRa/LSC border falling into *rpl*23 gene, an overall plastome size of 152,288-154,686 bp, and chromosome numbers of 2n=20 were found in clade A; whereas dorsally compressed and pubescent mericarps with filiform dorsal ribs, winged lateral ribs, numerous vittae in commissure and each furrow, IRa/LSC border falling into the *ycf*2 gene, an overall plastome size of 146,718-147,592 bp, and chromosome numbers of 2n=22 were discovered in clade B. Therefore, we established two new genera (*Shanopeucedanum* gen. nov. and *Sinopeucedanum* gen. nov.) to respectively accommodate the taxa of clades A and B. Furthermore, molecular dating analysis showed that the diversification of clades A and B occurred in the early Pleistocene and late Pliocene, respectively, which may have been driven by the complex geological and climate shifts of these periods. In summary, our study impelled a revision of *Peucedanum* s.l. members and improved the taxonomic system of the Apiaceae family.

## 1 Introduction

With the advancements of molecular phylogenetics, taxonomists conducted extensively taxonomic revisions in angiosperms and obtained the relatively stable system for orders and families, (Angiosperm Phylogeny Group, 2003, 2009, 2016; Bremer et al., 1998), as well as for those large genera with economic, medicinal and horticultural value (Miller et al., 2014; Li et al., 2012; Razafimandimbison et al., 2014; Drew et al., 2017; Xu and Hong, 2021; Fu et al., 2022; Scatigna et al., 2022). Therefore, molecular phylogenetics provides strong evidence for taxa that are difficult to morphologically identify.

*Peucedanum* s.l., the backbone member of the subfamily Apioideae, includes 100-120 species. Of these, forty species are distributed in China with 33 of them endemic (Sheh and Watson, 2005). The genus is widely distributed across Europe, Asia, and South Africa, with Europe and East Asia as the distribution centers (Wu et al., 2003; Spalik et al., 2004; Sheh and Watson, 2005; Zhang, 2011). *Peucedanum* s.l. can be identified by dorsally compressed mericarps with slightly prominent dorsal ribs, narrowly winged lateral ribs, and a broad commissure (Spalik et al., 2004; Sheh and Watson, 2005). However, members of this genus are heterogenous and always exhibit great variability in life-forms, leaf and fruit, and chemical constituents (Shneyer et al., 2003; Zhou et al., 2014; Wang et al., 2015; Ostroumova, 2018). For example, during our field botanical surveys, we found that four species (*P. dissolutum*, *P. guangxiense*, *P. mashanense* and *P. medicum*) have solitary or numerous stems that are solid, glabrous, and basally usually clothed with fibrous remnant sheaths, branched above; the leaves are basal and cauline, ternate or pinnate, and terminal leaflet diverse; the basal leaves are petiolate with petiole sheathing; the cauline leaves are reduced upwards with the leaf sheath expanded; inflorescences compound umbels, terminal and latera; the bracts are few or absent; the bracteoles are numerous and rarely few; the rays are numerous, unequal, and pubescent; the calyx teeth are conspicuous; and the petals are white and inflexed. Ten species (*P. ampliatum*, *P. formosanum*, *P. harry-smithii*, *P. harry-smithii* var. *grande*, *P. harry-smithii* var. *subglabrum*, *P. huangshanense*, *P. japonicum*, *P. praeruptorum*, *P. turgeniifolium* and *P. wawrae*) possessed stout rootstock, the crown of which was usually clothed with fibrous remnant sheaths; the stems were solitary and branched above; leaves were basal and cauline, ternate or pinnate, and the terminal leaflet was diverse; the basal leaves petiolate with petiole sheathing; the cauline leaves were reduced upwards and the leaf sheath expanded; inflorescences compound umbels, terminal and latera; the bracts were few or absent; the bracteoles were numerous, rarely few; the rays were unequal and pubescent; the calyx teeth were short or obsolete; and the petals were usually white, occasionally purple, and inflexed. Therefore, it noticed that the genus is a taxonomically confused group (Downie et al., 2010).

In addition, all molecular phylogenetic studies on *Peucedanum* s.l. have failed to recover this genus as a monophyletic group, of which only a few European species, with one species (*P. morisonii* Besser ex Schult.) extended to East Asia, were consistently clustered with *P. officinale* L., the type species of this genus (Downie et al., 2000; Spalik et al., 2004; Valiejo-Roman et al., 2006; Feng et al., 2009; Zhou et al., 2009, 2020; Lei et al., 2022; Liu et al., 2022). Therefore, *Peucedanum* s.s. was adopted (Kadereit and Bittrich, 2018) and several genera were restored or established to accommodate other European taxa, such as *Cervaria* Wolf, *Imperatoria* L., *Oreoselinum* Mill., *Pteroselinum* Rchb., *Thysselinum* Adans., *Xanthoselinum* Schur, *Holandrea* Reduron, Charpin & Pimenov, and *Taeniopetalum* Vis (Reduron et al., 1997; Ostroumova et al., 2016). Winter et al. (2008) also revised the African taxa of *Peucedanum* s.l. by establishing three new genera (*Afrosciadium* P.J.D. Winter, *Nanobubon* Magee, and *Notobubon* B.-E. van Wyk) and transferring 24 species into the *Afroligusticum* C. Norman, *Cynorhiza* Eckl. & Zeyh., and *Lefebvrea* A. Rich. genera based on morphological characteristics and molecular evidence. For Asian taxa previously residing in the *Peucedanum* s.l., genera*Kitagawia* Pimenov (Pimenov, 1986), *Haloselinum* Pimenov (Pimenov and Ostroumova, 2012), and *Sillaphyton* Pimenov (Pimenov et al., 2016) were separated and several transfers have been performed (Pimenov, 2017; Xiao et al., 2017; Gou et al., 2020; Liu et al., 2023; Song et al., 2023). However, classifications of numerous taxa have still not been satisfactorily resolved, especially for those endemic to China, such as *P. dissolutum*, *P. guangxiense*, *P. mashanense*, *P. medicum*, *P. ampliatum*, *P. formosanum*, *P. harry-smithii*, *P. harry-smithii* var. *grande*, *P. harry-smithii* var. *subglabrum*, *P. huangshanense*, *P. japonicum*, *P. praeruptorum*, *P. turgeniifolium*, and *P. wawrae*. Therefore, it is necessary and urgent to obtain a robust phylogenetic framework to investigate their classifications.

Currently, genomic-level data have begun to replace one or a few fragments for the phylogenetic analyses of plants (Ji et al., 2022). Genome skimming is an effective approach to obtain complete plastome and nrDNA sequences (Straub et al., 2012) and has been extensively and successfully used to solve the phylogeny of plants, especially for the taxonomically difficult and controversial taxa (Ruhsam et al., 2015; Ji et al., 2019, 2022; Li et al., 2019; Liu et al., 2021; Schneider et al., 2021; Choi et al., 2022; Sielemann et al., 2022; Cao et al., 2023; Cui et al., 2023; Lima et al., 2023; Peng et al., 2023; Song et al., 2023; Song et al., 2024a, 2024b). In previously published study (Liu et al., 2022), we also employed the genome skimming approach to generate plastome data to investigate the phylogenetic positions of *Peucedanum* s.l. members, which significantly improved the taxonomic understanding of this group. However, sampling of this genus was too small to completely aid the taxonomic revision of *Peucedanum* s.l.

Here, we newly generated nine high-quality plastomes and 43 nrDNA sequences of *Peucedanum* s.l. using the genome skimming approach. Together with molecular data previously reported by us, 103 plastomes and 43 nrDNA sequences were used in the phylogenetic analyses, including 21 out of the 33 *Peucedanum* s.l taxa endemic to China. Based on the phylogeny of large data sets, we aim to (1) test whether the previous taxonomic revisions of *Peucedanum* s.l species were reasonably given; (2) uncover the phylogenetic positions of taxa endemic to China, and combine evidence from plastome features, chromosome number, and morphological features to conduct taxonomic revisions; (3) explore historical diversification for core clades of endemic taxa.

## 2 Materials and methods

### 2.1 Taxa sampling, DNA extracting, and genome skimming

In this study, we collected nine *Peucedanum* s.l. taxa in the wild, including eight taxa endemic to China and one representative of *Peucedanum* s.s., *P. morisonii*. Then, the fresh young basal leaves were immediately stored in silica gel and dried for further DNA extraction. Vouchers were deposited in the herbarium of Sichuan University (Chengdu, China) (**Supplementary Table S1**). In addition, we also obtained root tips from the living plants to count chromosome number, except for *P. guangxiense* R.H. Shan & M.L. Sheh, *P. morisonii*, and *Semenovia malcolmii* (Hemsl. & H. Pearson) Pimenov (*P. torilifolium* H. Boissieu). Furthermore, the root tips of *P. mashanense* R.H. Shan & M.L. Sheh and *P. medicum* Dunn were obtained from our previous collection (Liu et al., 2022). Finally, we collected raw data from genome skimming within the Apiaceae subfamily Apioideae to recover 34 nrDNA sequences (**Supplementary Table S2**).

The total DNA of the newly collected samples was isolated from the silica-gel-dried leaves using the modified CTAB method (Doyle and Doyle, 1987). Subsequently, pair-end libraries with an average insert size of 300-400 bp were constructed according to the manufacturer’s protocol (Illumina, San Diego, CA, USA). Prepared libraries were sequenced on the Illumina NovaSeq platform at Personalbio (Shanghai, China). About 10 Gb of raw data for each sample were generated by genome skimming and then the raw data were filtered by software fastP v0.15.0 (-n 10 and -q 15) (Chen et al., 2018) to obtain high-quality reads.

### 2.2 Assembly and annotation of plastome and nrDNA

Based on the high-quality reads, we used GetOrganelle pipeline v1.7.5.0 (Jin et al., 2020) to assemble the complete plastomes with the default parameters and the *rbc*L sequence of *P. japonicum* (JF943288) as the seed. Furthermore, GetOrganelle pipeline v1.7.5.0 (Jin et al., 2020) was also employed to recover nrDNA sequences, setting the published nrDNA sequence of *P. japonicum* (KX757777) as reference. The assembled plastomes were initially annotated using the web server CPGAVAS2 (http://www.herbalgenomics.org/cpgavas2) (Shi et al., 2019). Then, the start and stop codons and intron positions were manually corrected in Geneious v9.0.2 (Kearse et al., 2012). For the annotation of the nrDNA, newly recovered sequences were compared with the reference in Geneious v9.0.2 (Kearse et al., 2012) to determine the borders of the ribosomal RNA genes (18S, 5.8S, and 26S ribosomal RNA genes).

### 2.3 Phylogenetic analyses

A total of 103 plastomes (40 *Peucedanum* species are distributed in China with 33 sampled in this study, the coverage of the current samplings accounting for 75%) and 43 nrDNA sequences of the Apiaceae subfamily Apioideae were used to reconstruct the phylogenetic trees (**Supplementary Table S3**). Of these sequences, nine plastomes were newly sequenced and 94 were downloaded from the National Center for Biotechnology Information (NCBI) that were previously reported by our team, and all 43 nrDNA sequences were newly generated. Among them, *Chamaesium mallaeanum* Farille & S.B. Malla and *Chamaesium viridiflorum* (Franch.) H. Wolff ex R.H. Shan were chosen as the outgroup according to the phylogenetic result of Wen et al. (2021). The 43 nrDNA sequences were aligned using MAFFT v7.221 (Katoh and Standley, 2013) to generate the matrix. For the plastome data, 79 commonly shared plastid protein-coding genes (PCGs) were manually extracted in Geneious v9.0.2 (Kearse et al., 2012) and respectively aligned using MAFFT v7.221 (Katoh and Standley, 2013), trimmed by trimAI (Capella-Gutierrez et al., 2009) and then the alignments were concatenated into a super matrix using PhyloSuite v1.2.2 (Zhang et al., 2020).

Two matrixes were subjected to maximum likelihood (ML) and Bayesian inference (BI) analyses. The ML analyses were performed using RAxML v8.2.8 (Stamatakis, 2014) with the GTRGAMMA model, and 1000 bootstrap replicates were used to estimate the support value (BS) for each node. For the BI analyses, phylogenetic trees were generated with MrBayes v3.2.7 (Ronquist et al., 2012). The best-fit substitution model (GTR+I+G) for the matrixes of the concatenated plastid protein-coding genes and nrDNA was determined using Modeltest v3.7 (Posada and Crandall, 1998). Two simultaneous and independent Markov chain Monte Carlo (MCMC) runs with 10 million generations were performed, sampling every 1000 generations. The runs were finished when the average standard deviation of the split frequencies was below 0.01. The first 25% of the trees were abandoned as burn-in and the remainders were used to generate the consensus tree and calculate posterior probabilities (PP). Finally, the phylogenetic trees were edited using FigTree v1.4.2 (Rambaut and Drummond, 2015).

### 2.4 Molecular dating

According to two pollen fossils within Apiaceae and the strategy adopted by Banasiak et al. (2013) and Wen et al. (2020, 2021), two calibration points constrained to a lognormal distribution were used to constrain the phylogenetic treeL (i) The stem node of Bupleureae was constrained with the lower bound of 33.90 Mya and the upper bound of 58.71 Mya; (ii) whereas the stem node of Pleurospermeae was calibrated with the lower bound of 33.90 Mya and the upper bound of 55.80 Mya. The standard deviation of the lognormal distribution was set to 0.5 for both calibration points. Based on the concatenated data set of 79 plastid protein-coding genes, the divergence times were estimated using BEAST v1.10.4 (Suchard et al., 2018) under the uncorrelated lognormal relaxed clock model, with a Yule speciation process and the best-fit nucleotide substitution model (GTR+I+G) detected using Modeltest v3.7 (Posada and Crandall, 1998). The ML tree inferred from the matrix of concatenated plastid protein-coding genes was used to fix topology. Two MCMC runs of 200,000,000 generations were performed, sampling every 20,000 generations. The convergence of running was checked using Tracer v1.7 (Rambaut et al., 2018), with an effective sample size (ESS) of all parameters not less than 200. The trees generated by the two runs were combined using LogCombiner v1.10.4 (Suchard et al., 2018) after discarding the first 20% of trees as burn-in, and then used to produce the maximum clade credibility tree with median ages and 95% highest posterior density (HPD) intervals with TreeAnotator v2.1.2 (Suchard et al., 2018). The result was exhibited in FigTree v1.4.2 (Rambaut and Drummond, 2015).

### 2.5 Chromosome counting

The chromosome number varies greatly between genera and species in the Apiaceae family, which is often used as one of the main evidences for exploring the intergeneric relationships, interspecific relationships, and species evolution of Apiaceae plants (Pan and Qin, 1981; Solov’eva et al., 1985; Ci, 1994; Jiang et al., 2002; Marhold, 2006; Pu et al., 2006; Zhang et al., 2006; Marhold et al., 2009; Kumar and Singhal, 2011; Sun et al., 2020; Liu et al., 2023). For example, previous studies reported that the basic chromosome number of the genus *Bupleurum* L. in China was 4, 6, 7, and 8, indicating that *Bupleurum* L. is a multi-base genus (Pan and Qin, 1981; Jiang et al., 2002; Liang et al., 2013). Researchers have shown that the chromosome number was different between species in the genus *Pleurospermum* Hoffm. in the Hengduan Mountains (HDM), with basic chromosome numbers of 9 and 11, which provided vital evidence for interspecific classification within the genus (Pan and Qin, 1981; Marhold, 2006; Pu et al., 2006; Kumar and Singhal, 2011; Sun et al., 2020). Zhang et al. (2006) analyzed the chromosome numbers and karyotypes of three populations of *Hansenia forbesii* (H. Boissieu) Pimenov & Kljuykov and found significant differences in the karyotype composition of the three populations, indicating that the polymorphism of karyotype composition among the *H. forbesii* populations was quite obvious, which was likely the result of adaptation to different environments. Ci (1994) found that the karyotype of *Saposhnikovia divaricata* was 2n=16, which can easily distinguish it from its related genera. Therefore, we collected the root tips of eight *Peucedanum* s.l. taxa in the current study. All the root tips were pre-treated with a solution of 2mM 8-hydroxyquinoline - 0.2% colchicine (1:1) for 4 h, fixed in Carnoy I (glacial acetic acid–absolute ethanol = 1:3) for 2 h, macerated in 1 N HCl at 60°C for 10 min, and squashed in carbol fuchsin. Finally, we counted the chromosome number under a light microscope (Olympus-BX51).

## 3 Results

### 3.1 Features of the plastomes and nrDNA

A total of nine high-quality plastomes were newly assembled in the current study. All of them exhibited a typical quadripartite structure, including a pair of inverted repeat regions (IRs: 12,455-25,821 bp) separated by a large single copy region (LSC: 86,721-100,043 bp) and a small single copy region (SSC: 16,080-17,728 bp). The overall sizes of the nine plastomes varied from 142,433 bp to 154,686 bp and the total GC content of which was 37.4%-37.6%. These plastomes encoded 112-113 unique genes, including 79 protein-coding genes, 29-30 tRNA genes, and 4 rRNA genes (**Table 1**). The *trn*T-GGU gene was lost in *P. praeruptorum* Dunn and *P. wawrae* (H. Wolff) S.W. Su ex M.L. Sheh. In addition, we also recovered 43 nrDNA sequences, including 18S, ITS1, 5.8S, ITS2, and 26S regions, with sequence length varied from 5,195 bp to 5,822 bp.

**Table 1:** Plastome features of nine *Peucedaum* s.l. taxa.

Taxon	Total length (bp)	LSC (bp)	SSC (bp)	IR (bp)	Total GC content (%)	Total genes (unique)	Protein coding genes (unique)	rRNA genes (unique)	tRNA genes (unique)
*P. dissolutum*	154,686	86,964	16,080	25,821	37.4	113	79	4	30
*P. guangxiense*	152,969	86,721	17,728	24,260	37.5	113	79	4	30
*P. harry-smithii*	147,197	92,400	17,521	18,638	37.6	113	79	4	30
*P. harry-smithii* var. *subglabrum*	147,143	92,469	17,522	18,576	37.6	113	79	4	30
*P. huangshanense*	147,437	92,501	17,580	18,678	37.6	113	79	4	30
*P. morisonii*	147,105	93,594	17,537	17,987	37.6	113	79	4	30
*P. praeruptorum*	146,954	92,011	17,521	18,711	37.6	112	79	4	29
*P. wawrae*	147,179	92,121	17,522	18,768	37.6	112	79	4	29
*Semenovia malcolmii* (*P. torilifolium*)	142,433	100,043	17,480	12,455	37.4	113	79	4	30

### 3.2 Phylogenetic analyses and divergence time estimation

**Figure 1:**
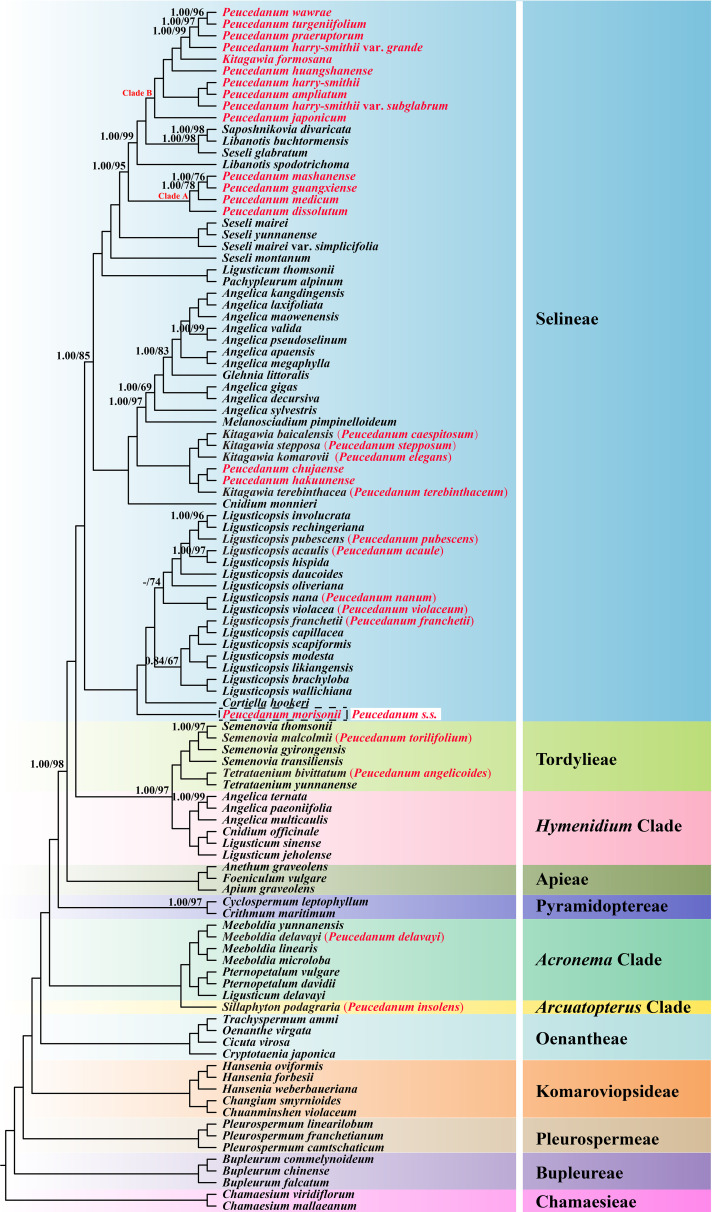
Phylogenetic topologies based on concatenated plastid protein-coding genes inferred by the Bayesian inference (BI) and maximum likelihood (ML) methods. The numbers represent Bayesian posterior probabilities (PP) and maximum likelihood bootstrap values (BS). Nodes with PP=1.00/BS=100 were not displayed. – denotes values < 0.50/50.

The phylogenetic topologies from the ML and BI analyses based on concatenated plastid protein-coding genes were identical. All the tribes or clades involved in the current study with more than one sample were recovered as a well-supported monophyletic group. However, the monophyly of *Peucedanum* s.l. failed to recover and members of this genus scattered in four tribes (or clades): (1) *Sillaphyton podagraria* (H. Boissieu) Pimenov (*P. insolens* Kitag.) was located in the *Arcuatopterus* clade (PP = 1.00, BS = 100). (2) *Meeboldia delavayi* (Franch.) W. Gou & X.J. He (*P. delavayi* Franch.) was nested in the *Meeboldia* H. Wolff genus belonging to the *Acronema* clade (PP = 1.00, BS = 100). (3) *Semenovia malcolmii* (Hemsl. & H Pearson) Pimenov (*P. torilifolium* H. Boissieu) was placed in *Semenovia* Regel & Herder, while *Tetrataenium bivittatum* (H. Boissieu) Manden. (*P. angelicoides* H. Wolff ex Kretschmer) was sister to *Tetrataenium yunnanense* (Franch.) Manden. ex Q.Y. Xiao & X.J. He, belonging to Tordylieae (PP = 1.00, BS = 100). (4) The remainders were included in Selineae (PP = 1.00, BS = 100). Although most of the members of *Peucedanum* s.l. fell into the Selineae tribe, these taxa have not been grouped in a unit instead of five clades: (i) *P. morisonii* Besser ex Schult., the representative of *Peucedanum* s.s., solely represented a clade and was sister to the clade formed by *Cortiella hookeri* (C.B. Clarke) C. Norman and *Ligusticopsis* Leute genus (PP = 1.00, BS = 100); (ii) five species, *Ligusticopsis acaulis* (R.H. Shan & M.L. Sheh) Pimenov (*P. acaule* R.H. Shan & M.L. Sheh), *Ligusticopsis franchetii* (C.Y. Wu & F.T. Pu) B.N. Song, C.K. Liu & X.J. He (*P. franchetii* C.Y. Wu & F.T. Pu), *Ligusticopsis nana* (R.H. Shan & M.L. Sheh) C.K. Liu & X.J. He (*P. nanum* R.H. Shan & M.L. Sheh), *Ligusticopsis pubescens* (Hand.-Mazz.) J.J. Deng, C.K. Liu & X.J. He (*P. pubescens* Hand.-Mazz.), and *Ligusticopsis violacea* (R.H. Shan and M.L. Sheh) C.K. Liu & X.J. He (*P. violaceum* R.H. Shan & M.L. Sheh) were nested in the *Ligusticopsis* genus with high support (PP = 1.00, BS = 100); (iii) *Kitagawia baicalensis* (Redowsky ex Willd.) Pimenov (*P. baicalense* (Redowsky ex Willd.) W.D.J. Koch), *Kitagawia komarovii* Pimenov (*P. elegans* Kom.), *Kitagawia stepposa* (Y.H. Huang) Pimenov (*P. stepposum* Y.H. Huang), *Kitagawia terebinthacea* (Fisch. ex Trevir.) Pimenov (*P. terebinthaceum* (Fisch. ex Trevir.) Turcz.), *P. chujaense* K. Kim, S.H. Oh, Chan S. Kim & C.W. Park, and *P. hakuunense* Nakai were clustered into a clade (PP = 1.00, BS = 100); (iv) four species [*P. dissolutum* (Diels) H. Wolff, *P. guangxiense* R.H. Shan & M.L. Sheh, *P. mashanense* R.H. Shan & M.L. Sheh, and *P. medicum* Dunn] formed clade A (PP = 1.00, BS = 100); (v) the remaining ten taxa (*P. ampliatum*, *P. formosanum*, *P. harry-smithii*, *P. harry-smithii* var. *grande*, *P. harry-smithii* var. *subglabrum*, *P. huangshanense*, *P. japonicum*, *P. praeruptorum*, *P. turgeniifolium*, and *P. wawrae*) constituted clade B with strong support (PP = 1.00, BS = 100) (**Figure 1**). These five clades were also recovered in the nrDNA tree, whereas *P. huangshanense* Lu Q.Huang, H.S. Peng & S.S. Chu was not located in clade B but was sister to *Pachypleurum alpinum* Ledeb. and then nested into *Kitagawia* Pimenov (**Supplementary Figure S1**).

**Figure 2:**
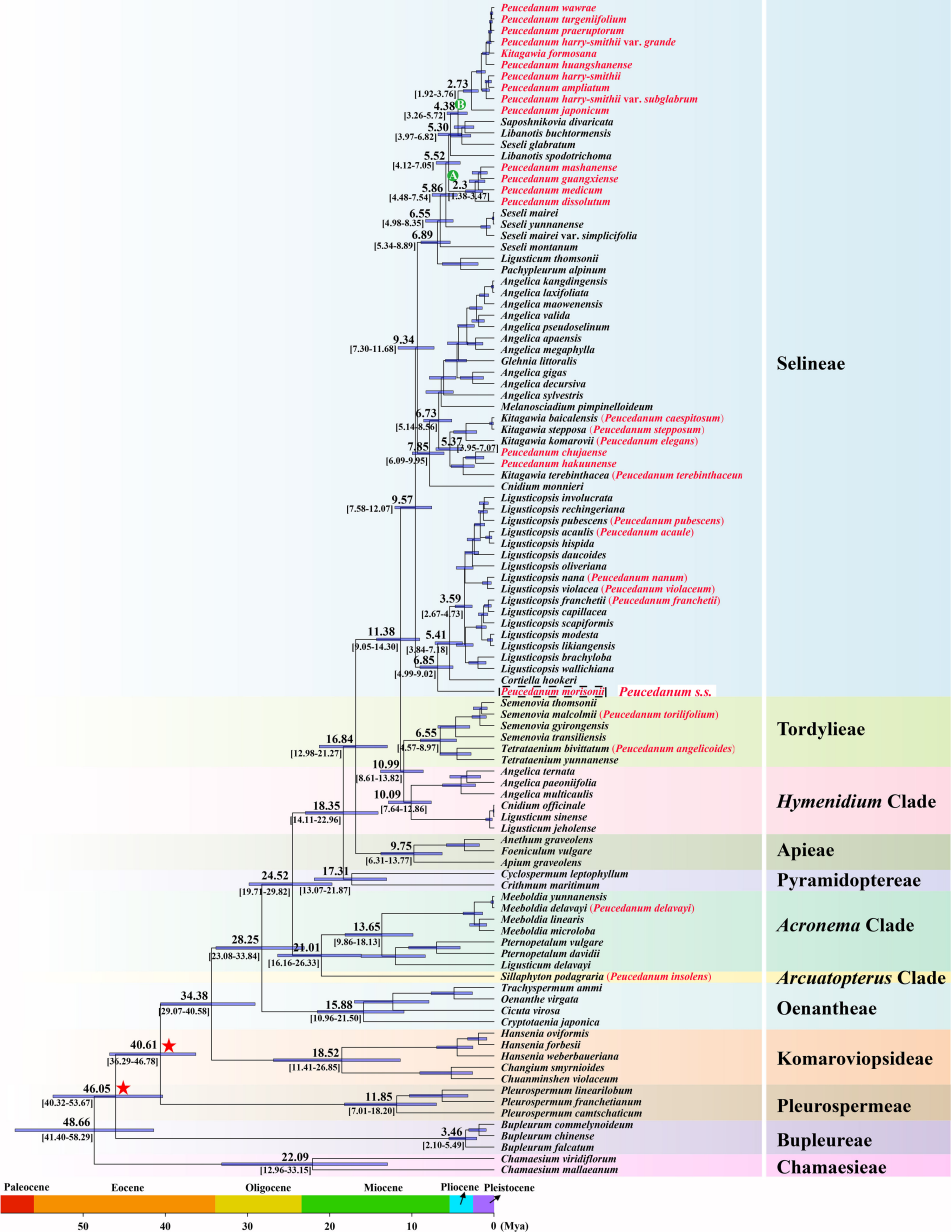
Divergence time estimation based on concatenated plastid protein-coding genes. Numbers above/under branches represent mean divergent age and 95% highest posterior density interval. Red stars indicate the calibration points for the molecular dating.

In addition, molecular dating analysis based on the ML tree of concatenated plastid protein-coding genes was performed (**Figure 2**). The molecular dating results indicated that the crown age of the Selineae tribe was estimated to be 9.75 Mya (95% HPD: 12.07-7.58 Mya). Within this tribe, the diversification of clades A and B occurred at 2.30 Mya (95% HPD: 3.47-1.38 Mya) in the early Pleistocene period and 2.73 Mya (95% HPD: 3.76-1.92 Mya) in the late Pliocene period, respectively.

### 3.3 Chromosome numbers

The chromosome numbers of eight *Peucedanum* s.l. taxa were counted. Among them, the chromosome numbers of three species, *P. dissolutum* (Diels) H. Wolff, *P. mashanense* R.H. Shan & M.L. Sheh, and *P. medicum* Dunn, were 2n=20, while the chromosome numbers of the remainders were 2n=22 (**Supplementary Figure S2**).

## 4 Discussion

### 4.1 Phylogenetic framework and taxonomic implications

The phenomenon of incongruences between plastome-based and nrDNA-based phylogenies is common in Apiaceae (Xiao et al., 2017; Lei et al., 2022; Song et al., 2024a, b), and our study was no exception. In our study, the topologies generated from the PCGs and nrDNA were largely similar, except for the discordance regarding the position of *P. huangshanense*, which may be caused by incomplete lineage sorting due to the young evolutionary history (Wen et al., 2020, 2021). Same as all previous phylogenetic studies (Downie et al., 2000; Spalik et al., 2004; Valiejo-Roman et al., 2006; Feng et al., 2009; Zhou et al., 2009, 2020; Lei et al., 2022; Liu et al., 2022), phylogenetic analyses respectively based on concatenated plastid protein-coding genes and nrDNA sequences in the current study failed to recover *Peucedanum* s.l. as a monophyletic group. Specifically, both analyses robustly supported that *P. morisonii*, the representative of *Peucedanum* s.s., was distantly related to other *Peucedanum* s.l. members. The distant relationship was further justified by the morphological features: ternate leaves, linear leaflets, yellow petals, and dorsally compressed and glabrous mericarps with 1 vittae in each furrow and 2 on commissure, which were the recognized features of *Peucedanum* s.s (Kadereit and Bittrich, 2018), and significantly distinguished *P. morisonii* from other *Peucedanum* s.l. members. Hence, all these *Peucedanum* s.l. taxa were not “truly *Peucedanum* plants” and the taxonomic positions of them need to be re-explored.

**Figure 3:**
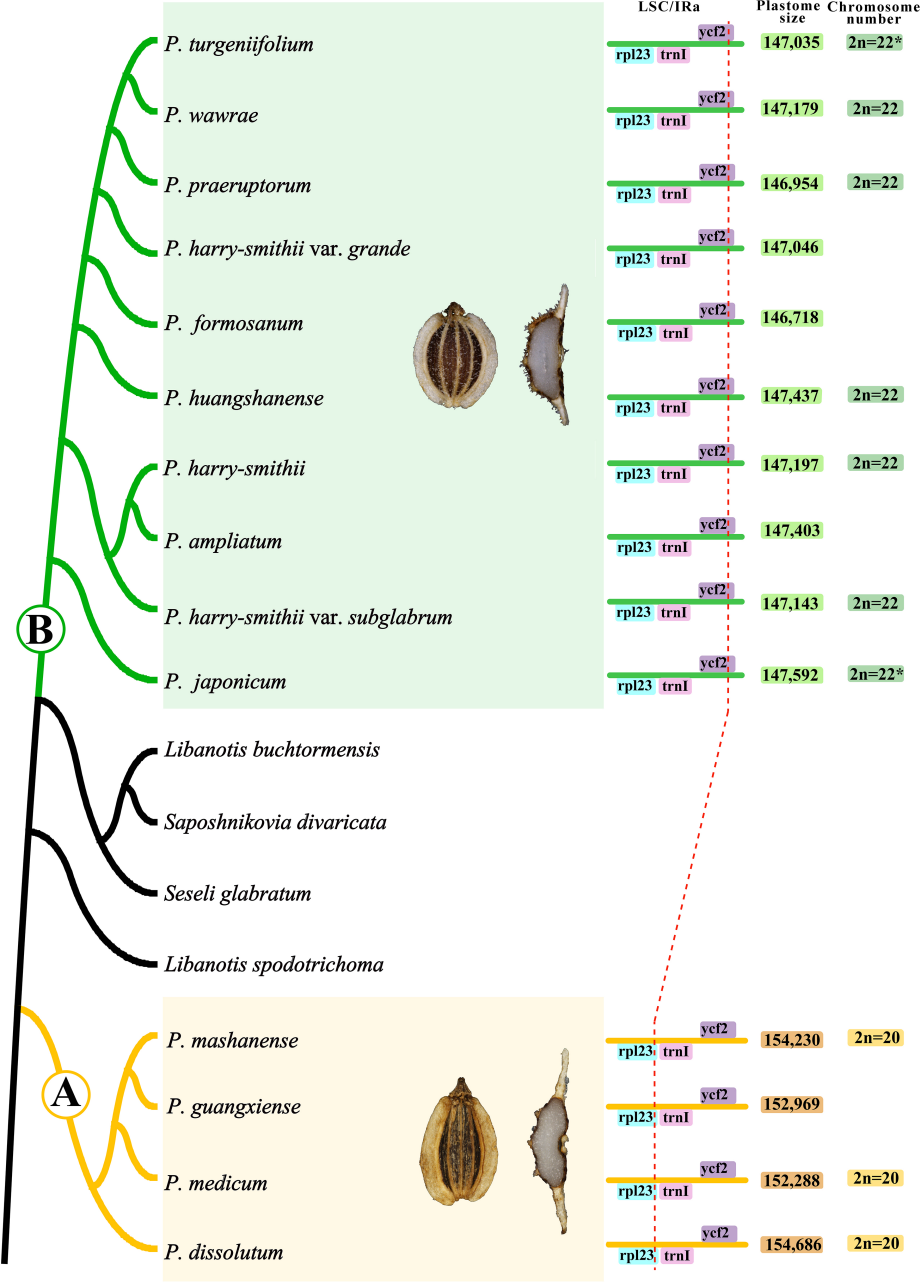
Comparisons of mericarps, plastomes, and chromosome number between clade A and clade B. The chromosome numbers for *P. turgeniifolium* and *P. japonicum* marked with a * were obtained from the results of Zhang et al. (2006) and Sun et al. (1996), respectively.

In addition, the PCG-based phylogenetic analyses showed that 14 *Peucedanum* s.l. taxa were divided into two monophyletic clades (clade A and clade B) by *Saposhnikovia divaricata* (Turcz.) Schischk., *Seseli glabratum* Willd. ex Schult., *Libanotis buchtormensis* (Fisch.) DC., and *L. spodotrichoma* K.T. Fu with robust supports. Furthermore, obvious morphological differences between 14 *Peucedanum* s.l. taxa and these species were also observed: mericarps with filiform dorsal ribs, winged lateral ribs, and numerous vittae in each furrow and commissure, easily distinguished the 14 *Peucedanum* s.l. taxa from the mericarps with fake vittae in each rib, and 1 vittae in each furrow and 2 on commissure of *Saposhnikovia divaricata*, from mericarps with equally filiform or keeled ribs, and 1 vittae in each furrow and 2 on commissure of *S. glabratum*, *L. buchtormensis* and *L. spodotrichoma* (Sheh, 1992; Sheh and Watson, 2005). Thus, it was clearly inappropriate to regard all these taxa as a unit, whether based on morphological features or phylogenetic analyses. Furthermore, we also detected that clades A and B had significant morphological differences, but the morphological characteristics within each clade were unified. In detail, four members of clade A were perennial herbs; stem solitary or numerous, solid, glabrous, basal usually clothed with fibrous remnant sheaths, branched above; leaves basal and cauline, ternate or pinnate, terminal leaflet diverse; basal leaves petiolate, petioles sheathing; cauline leaves reduced upwards, leaf sheath expand; inflorescences compound umbels, terminal and latera; bracts few or absent; bracteoles numerous, rarely few; rays numerous, unequal, pubescent; calyx teeth conspicuous; petals white, inflexed; mericarp dorsally compressed, glabrous; dorsal ribs filiform, lateral ribs narrowly winged; numerous vittae in commissure and each furrow. Ten members of clade B were perennial herbs; rootstock stout, crown usually clothed with fibrous remnant sheaths; stem solitary, branched above; leaves basal and cauline, ternate or pinnate, terminal leaflet diverse; basal leaves petiolate, petioles sheathing; cauline leaves reduced upwards, leaf sheath expand; inflorescences compound umbels, terminal and latera; bracts few or absent; bracteoles numerous, rarely few; rays unequal, pubescent; calyx teeth short or obsolete; petals usually white, occasionally purple, inflexed; mericarp dorsally compressed, pubescent; dorsal ribs filiform, lateral ribs narrowly winged; numerous vittae in commissure and each furrow. These unique features of clades A and B can also easily distinguish them from other related genera. Meanwhile, the plastome structure and chromosome numbers of clades A and B were also different, such as the IRa/LSC border falling into the *rpl*23 gene, an overall plastome size of 152,288-154,686 bp, and chromosome numbers of 2n=20 in clade A, and the IRa/LSC border falling into the *ycf*2 gene, an overall plastome size of 146,718-147,592 bp, and chromosome numbers of 2n=22 in clade B (**Figure 3**). Therefore, we established two new genera (*Shanopeucedanum* gen. nov. and *Sinopeucedanum* gen. nov.) to respectively accommodate the taxa of clades A and B and the treatment was accepted based on morphology, plastomes, chromosome number, and phylogeny.

Our PCG-based phylogenies also resolved the positions of the remaining *Peucedanum* s.l. species with high support. Six species formed a monophyletic clade, in which four species (*P. baicalense*, *P. elegans*, *P. stepposum*, and *P. terebinthaceum*) have been transferred into the *Kitagawia* genus (Pimenov, 1986, 2017) and we suggested that the remaining two species (*P. chujaense* and *P. hakuunense*) also should be transferred into the *Kitagawia* genus. Five species (*P. acaule*, *P. franchetii*, *P. nanum*, *P. pubescens*, and *P. violaceum*) were nested in *Ligusticopsis* and have been merged into this genus (Pimenov, 2017; Deng et al., 2022; Liu et al., 2023; Song et al., 2023). *P. insolens* formed a separate clade and a monotypic genus, *Sillaphyton*, was established by Pimenov to accommodate this species (Pimenov et al., 2016). *P. delavayii* and *P. torilifolium* respectively nested in *Meeboldia* and *Semenovia*, while *P. angelicoides* was sister to *Tetrataenium yunnanense*, and they have been transferred into *Meeboldia*, *Semenovia*, and *Tetrataenium*, respectively (Xiao, 2017; Xiao et al., 2017; Gou et al., 2020). Hence, with high support and extended samples, our results strongly supported the previous taxonomic revisions for the above species and provide new additional evidence to accept these treatments.

### 4.2 Estimation divergence time

The diversification of tribes within the Apiaceae family has been inferred based on robust phylogenetic frameworks obtained from transcriptome and plastome datasets by Wen et al. (2020, 2021). However, limited samples for each tribe were employed in both studies, such as only five species of the Selineae tribe in transcriptome datasets and fourteen species of the Selineae tribe in plastome datasets, which may have led to inaccurate results. Due to the majority of the *Peucedanum* s.l. taxa falling into Selineae, we extended the samples of this tribe to explore the molecular dating analysis in the current study. Our result showed that the diversification of the Selineae tribe occurred at 9.75 Mya (95% HPD: 12.07-7.58 Mya), which was slightly earlier than 5.32 Mya (95% HPD: 7.25-3.60 Mya) and 7.70 Mya (95% HPD: 10.09-5.74 Mya) inferred by Wen et al. (2020, 2021). With more samples, and therefore, more accurate results, we justified that the Selineae tribe was a young lineage and the members of this tribe were the results of recent diversification.

Within the Selineae tribe, the diversification of clades A and B occurred at 2.30 Mya (95% HPD: 3.47-1.38 Mya) (around the early Pleistocene) and 2.73 Mya (95% HPD: 3.76-1.92 Mya) (around the late Pliocene), respectively. Previous studies have proven that the dramatic uplift of Qinhai-Tibet Plateau (QTP) occurred from the late Miocene to the early Pliocene (Harrison et al., 2001; Royden et al., 2008), which significantly modified the global climate (An et al., 2001; Shi et al., 2005), and at least four major glaciations took place in East Asia during the Pleistocene (Shi, 1998). These complex geological and climate shifts drove the expansion/contraction of the species range and isolation in East Asia and thereby facilitated species radiation (Axelrod et al., 1998; Li and Fang, 1999; Qiu et al., 2011), which is thought to have facilitated the diversification of wide spectrum of East Asia plants (Qian and Ricklefs, 2000; Wu et al., 2007; Wen et al., 2014; Favre et al., 2015; Ji et al., 2019) and thus could also have caused isolation and drove species diversification in clades A and B. Thus, we speculate that the diversification in clades A and B may have been driven by complex geological and climate shifts.

### 4.3 Taxonomic treatments

***Shanopeucedanum* B.N.Song, C.K. Liu & X.J. He, gen. nov.**

Type: *Shanopeucedanum medicum* (Dunn) C.K. Liu & X.J. He.

Diagnosis: The genus can be easily distinguished from *Peucedanum* s.s. and related genera by white petals, dorsally compressed and glabrous mericarps with filiform dorsal ribs, winged lateral ribs, and numerous vittae in commissure and each furrow.

Description: Perennial herbs. Stem solitary or numerous, solid, glabrous, basal usually clothed with fibrous remnant sheaths, branched above. Leaves basal and cauline, ternate or pinnate, terminal leaflet diverse; basal leaves petiolate, petioles sheathing; cauline leaves reduced upwards, leaf sheath expanded. Inflorescences compound umbels, terminal and latera; bracts few or absent; bracteoles numerous, rarely few; rays numerous, unequal, pubescent; calyx teeth conspicuous; petals white, inflexed. Mericarp dorsally compressed, glabrous; dorsal ribs filiform, lateral ribs narrowly winged; numerous vittae in commissure and each furrow. 2n=20.

Diversity: Four species.

Distribution: Endemic to China.

Key to species of *Shanopeucedanum*.

1. Stem solitary; leaves ternate, rarely pinnate*……………………*

*  ……………………………………..Shanopeucedanum medicum.*

1. Stem numerous, leaves pinnate.

2. Terminal leaflet rhombic.......…………….…………………

   ……………………….…….*Shanopeucedanum mashanense*.

2. Terminal leaflet ovate.

 3. Mericarp large; leaves 3-pinnate.…………………….…

   …………………………….*Shanopeucedanum dissolutum*.

 3. Mericarp small; leaves 2-pinnate……………….….……

   ……………………………*Shanopeucedanum guangxiense*.

*Shanopeucedanum dissolutum* (Diels) C.K. Liu & X.J. He, comb. nov.

≡*Peucedanum dissolutum* (Diels) H. Wolff, Repert. Spec. Nov. Regni Veg. 21: 247. 1925.

≡*Angelica dissoluta* Diels, Bot. Jahrb. Syst. 29: 499. 1900.

Type: CHINA. Sichuan, Nanchuan, Kên ao p’ing, Abhänge, von Rosthorn 659 (syntype: O 2014068).

*Shanopeucedanum guangxiense* (R.H. Shan & M.L. Sheh) C.K. Liu & X.J. He, comb. nov.

≡*Peucedanum guangxiense* R.H. Shan & M.L. Sheh, Acta Phytotax. Sin. 24: 308. 1986.

Type: CHINA. Guangxi, Jingxi, 300 m alt., 8 November 1961, Wu Xinfang 23493 (holotpye: NAS 00082770).

*Shanopeucedanum mashanense* (R.H. Shan & M.L. Sheh) C.K. Liu & X.J. He, comb. nov.

≡*Peucedanum mashanense* R.H. Shan & M.L. Sheh, Acta Phytotax. Sin. 24: 304. 1986.

Type: CHINA. Guangxi, Mashan, 300 m alt., 7 September 1958, Zhong Shuquan 301547 (holotype: IBSC; isotype: KUN 0566764).

*Shanopeucedanum medicum* (Dunn) C.K. Liu & X.J. He, comb. nov.

≡*Peucedanum medicum* Dunn, J. Linn. Soc. Bot. 35: 496.1903.

Type: CHINA. Hupeh, Ichang and intermediate neighborhood, February 1887, Henry 15462006 (lectotpye, designated by Pimenov (2017): K 000685413, 000685414; isolectotype: BM, E, K 000685416, NY 00406177, P 00752971); Hupeh, Nam-To, 1887, Henry 1906 (syntype: K 000685415); Hupeh, Fang, Henry 5868A (syntypes: K 000685417, LE, NY 00406176, P 00752968); Szechuen, South Wushan, Henry 7473 (syntypes: LE, P 02272028).

***Sinopeucedanum* B.N.Song, C.K. Liu & X.J. He, gen. nov.**

Type: *Sinopeucedanum harry-smithii* (Fedde ex H. Wolff) C.K. Liu & X.J. He.

Diagnosis: The new genus can be easily recognized from *Peucedanum* s.s. and related genera by white or purple petals, dorsally compressed and pubescent mericarps with filiform dorsal ribs, winged lateral ribs, and numerous vittae in commissure and each furrow.

Description: Perennial herbs. Rootstock stout, crown usually clothed with fibrous remnant sheaths. Stem solitary, branched above. Leaves basal and cauline, ternate or pinnate, terminal leaflet diverse; basal leaves petiolate, petioles sheathing; cauline leaves reduced upwards, leaf sheath expanded. Inflorescences compound umbels, terminal and latera; bracts few or absent; bracteoles numerous, rarely few; rays unequal, pubescent; calyx teeth short or obsolete; petals usually white, occasionally purple, inflexed. Mericarp dorsally compressed, pubescent; dorsal ribs filiform, lateral ribs narrowly winged; numerous vittae in commissure and each furrow. 2n=22.

Diversity: Ten species.

Distribution: China, Japan, Korea, and Philippines.

Key to species of *Sinopeucedanum*.

1. Leaves ternate.

2. Calyx teeth inconspicuous.

 3. Umbels small; bracteoles linear…………………….……

  .………………………………….*Sinopeucedanum wawrae*.

 3. Umbels large; bracteoles pinnate and linear coexisted.…

  ………………………………*Sinopeucedanum ampliatum*.

2. Calyx teeth conspicuous.

 4. Anthers purple...…………………………………………

  ……………………….….*Sinopeucedanum huangshanense*.

 4. Anthers white.

  5. Stem glabrous…………………………….……………

  …………………………….*Sinopeucedanum japonicum*.

  5. Stem tomentose in upper parts.

   6. Bracteoles longer than flowers; fruit densely hispid

     ………………………..*Sinopeucedanum formosanum*.

   6. Bracteoles shorter than flowers; fruit sparsely

     puberulent…….*.Sinopeucedanum praeruptorum*.

1. Leaves pinnate.

7. Calyx teeth inconspicuous……………….…………………*Sinopeucedanum turgeniifolium*.

7. Calyx teeth conspicuous.

 8. Stem and leaves densely pubescent………………………..

  ……………………………....*Sinopeucedanum harry-smithii*.

 8. Stem and leaves glabrous or sparsely pubescent.

  9. Umbels small; mericarp suborbicular….……………….

   ……………………………...*Sinopeucedanum subglabrum*.

  9. Umbels large; mericarp obovate..………….……………

   …………………………………...*Sinopeucedanum grande*.

*Sinopeucedanum ampliatum* (K.T. Fu) C.K. Liu & X.J. He, comb. nov.

≡*Peucedanum ampliatum* K.T. Fu, Fl. Tsinling. 1(3): 462. 1981.

Type: CHINA. Shaanxi, Shanyang, Tianzhushan, 1660 m alt., 3 July 1964, Liang Yiming, Yang Jinxiang 3126 (holotype: WUK 228591).

*Sinopeucedanum formosanum* (Hayata) C.K. Liu & X.J. He, comb. nov.

≡*Peucedanum formosanum* Hayata, Icon. Pl. Formosan. 10: 22. 1921.

≡*Kitagawia formosana* (Hayata) Pimenov, Turczaninowia 20(2): 167. 2017.

Type: CHINA. Taiwan, Mt. Niitaka, ad 10000 ped. alt. [Nanto], 19 October 1906, Kawakami, Mori 2052 (holotype: TAIF).

*Sinopeucedanum grande* (K.T. Fu) C.K. Liu & X.J. He, comb. et stat. nov.

≡*Peucedanum harry-smithii* Fedde ex H. Wolff var. *grande* (K.T. Fu) R.H. Shan & M.L. Sheh, Fl. Reipubl. Popularis Sin. 55(3): 164. 1992.

≡*Peucedanum praeruptorum* Dunn var. *grande* K.T. Fu, Fl. Tsinling. 1(3): 428. 1981.

Type: CHINA. Shensi, Hwa-in Hsien, Hwa-shan, 1580 m alt., 5 August 1973, Fu Kuntsun 16884 (holotype: WUK).

*Sinopeucedanum harry-smithii* (Fedde ex H. Wolff) C.K. Liu & X.J. He, comb. nov.

≡*Peucedanum harry-smithii* Fedde ex H. Wolff, Repert. Spec. Nov. Regni Veg. 33: 247. 1933.

Type: CHINA. Shansi, Chieh-hsiuh-Distr. Sung-lin-miao, Cho mei shan, in prato apricot, ca. 1000 m alt., 3 October 1924, H. Smith 7609 (lectotype, designated by Pimenov (2017): UPS; isolectotypes: MO 150814, PE 00935536).

*Sinopeucedanum huangshanense* (Lu Q.Huang, H.S. Peng & S.S. Chu) C.K. Liu & X.J. He, comb. nov.

≡*Peucedanum huangshanense* Lu Q. Huang, H.S. Peng & S.S. Chu, Phytotaxa 430: 21. 2020.

Type: CHINA. Anhui, Mount Huangshan, on the forest margins and cliffs, ca. 1600 m alt., 20 August 2018, Peng 082011 (holotype: ACM; isotype: ACM).

*Sinopeucedanum japonicum* (Thunb.) C.K. Liu & X.J. He, comb. nov.

≡*Peucedanum japonicum* Thunb., Nova Acta Regiae Soc. Sci. Upsal. 4: 38. 1783.

≡*Anethum japonicum* (Thunb.) Koso-Pol., Bull. Soc. Imp. Nat. Mosc. n. s. 29: 117. 1916.

Type: JAPAN. San Bofu, vulgo Fama Bofu feu Iamma Bofu, i. e. Bofu littoralis Kaempf. Am. ex. Fafc. V. p. 825 (lectotype, designated by Thunb. (1784): UPS).

=*Peucedanum japonicum* Thunb. f. *album* Q.H. Yang & Q. Tian, Acta Bot. Boreal. Ocid. Sin. 28(2): 399. 2008.

Type: CHINA. Zhejiang, Zhoushan, 22 June 2007, Tian Qi, Yang Qinhua, Zhou Xiangyu 07-0331 (holotype: CSH 0067982).

*Sinopeucedanum praeruptorum* (Dunn) C.K. Liu & X.J. He, comb. nov.

≡*Peucedanum praeruptorum* Dunn, J. Linn. Soc. Bot. 35: 497. 1903.

≡*Kitagawia praeruptora* (Dunn) Pimenov, Turczaninowia 20(2): 167. 2017.

Type: CHINA. Hupeh, Ichang, 1887, Henry 2911 (syntypes: P 02272036, 02272037); Changyang, Henry 7505 (syntype: K); Szechuan, North Wushan, July 1888, Henry 7475 (lectotypes, designated by Pimenov (2017): E 00002618, 00002619; isolectotypes: BM 000885390, G, K 000685408, 000685409, 000685411, 000685412, P 022722038, 022722039).

*Sinopeucedanum subglabrum* (R.H. Shan & M.L. Sheh) C.K. Liu & X.J. He, comb. et stat. nov.

≡*Peucedanum harry-smithii* Fedde ex H. Wolff var. *subglabrum* (R.H. Shan & M.L. Sheh) R.H. Shan & M.L. Sheh, Fl. Reipubl. Popularis Sin. 55(3): 164. 1992.

≡*Peucedanum hirsutiusculum* (Y.C. Ma) R.H. Shan & M.L. Sheh var. *subglabrum* R.H. Shan & M.L. Sheh, Acta Phytotax. Sin. 24(4): 310. 1986.

Type: CHINA. Henan, Song Xian, 1000 m alt., 15 September 1965, Wu Peigeng, Wang Wanli 651161 (holotype: NAS 00026834; isotype: NAS 00042627).

*Sinopeucedanum turgeniifolium* (H. Wolff) C.K. Liu & X.J. He, comb. nov.

≡*Peucedanum turgeniifolium* H. Wolff, Act. Hort. Gothob. 2: 323. 1926.

Type: CHINA. Sichaun, Ch’osodjo, Ö om älven. Torr buskäng, 18 October 1922, H. Smith 4826 (lectotype, designated by Pimenov (2017): UPS; isolectotype: GB).

*Sinopeucedanum wawrae* (H. Wolff) C.K. Liu & X.J. He, comb. nov.

≡*Peucedanum wawrae* (H. Wolff) S.W. Su ex M.L. Sheh, Fl. Reipubl. Popularis Sin. 55(3): 149. 1992.

≡*Seseli wawrae* H. Wolff, Repert. Spec. Nov. Regni Veg. 27: 315. 1930.

Type: CHINA. Shandong, Chefoo, ravines in hills, 7 August 1920, Cowdry 757 (lectotype, designated by Pimenov (2017): K 000697461; isolectotype: K 000697460).

## 5 Conclusions

Based on the raw data generated from genome skimming, nine high-quality plastomes and 43 nrDNA sequences were presented. We performed phylogenetic analyses respectively based on concatenated plastid protein-coding genes and nrDNA sequences, including 30 members of *Peucedanum* s.l. with 21 endemic to China. Both analyses failed to recover *Peucedanum* s.l. as a monophyletic group and robustly supported that *P. morisonii*, the representative of *Peucedanum* s.s., was distantly related to other *Peucedanum* s.l. members, which implied that all these *Peucedanum* s.l. taxa were not “truly *Peucedanum* plants” and the distant relationship was also further supported by morphological evidence. Plastid-based phylogenies recognized two monophyletic clades, clade A (four species) and clade B (ten taxa). Furthermore, obvious recognized features from the morphology, plastomes, and chromosome number for each clade were detected, such as clade A possessing white petals, dorsally compressed and glabrous mericarps with filiform dorsal ribs, winged lateral ribs, and numerous vittae in commissure and each furrow; IRa/LSC border falling into the *rpl*23 gene; an overall plastome size of 152,288-154,686 bp; and chromosome numbers of 2n=20, while clade B possessed white or purple petals, dorsally compressed and pubescent mericarps with filiform dorsal ribs, winged lateral ribs, and numerous vittae in commissure and each furrow; IRa/LSC border falling into the *ycf*2 gene; an overall plastome size of 146,718-147,592 bp; and chromosome numbers of 2n=22. Therefore, we established two new genera (*Shanopeucedanum* and *Sinopeucedanum*) to accommodate the taxa of clades A and B, respectively. With high support and extended samples, our results also strongly support the previous taxonomic revisions for *Peucedanum* s.l. taxa. Furthermore, molecular dating analysis showed that the diversification of clades A and B occurred in the early Pleistocene and late Pliocene, respectively, which may have been driven by the complex geological and climate shifts in these periods.

## Data Availability

The datasets presented in this study can be found in online repositories. The names of the repository/repositories and accession number(s) can be found in the article/**Supplementary Material**.
