# Supplementary material for: Genome skimming provides evidence to accept two new genera (Apiaceae) separated from the Peucedanum s.l
Source: Front Plant Sci. 2025 Jan 20;15:1518418. doi: 10.3389/fpls.2024.1518418 (PMC11788392; doi:10.3389/fpls.2024.1518418)
Supplement: Supplementary file 1 [file DataSheet1.docx]

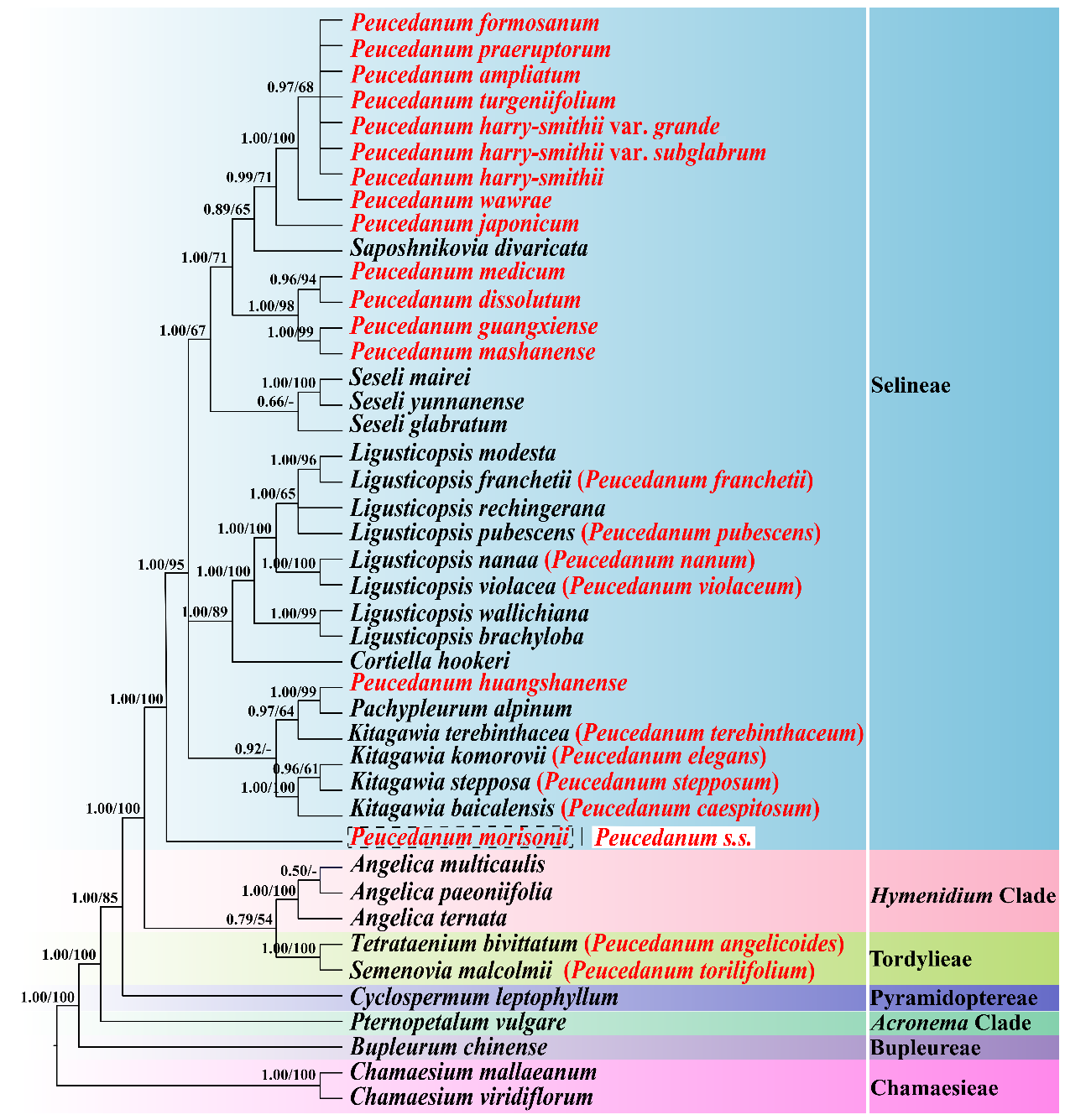


Figure S1. Phylogenetic topology based on nrDNA sequences inferring by Bayesian inference (BI) and Maximum likelihood (ML) methods. Numbers represent Bayesian posterior probabilities (PP) and maximum likelihood bootstrap values (BS). – means the values < 0.50/50.


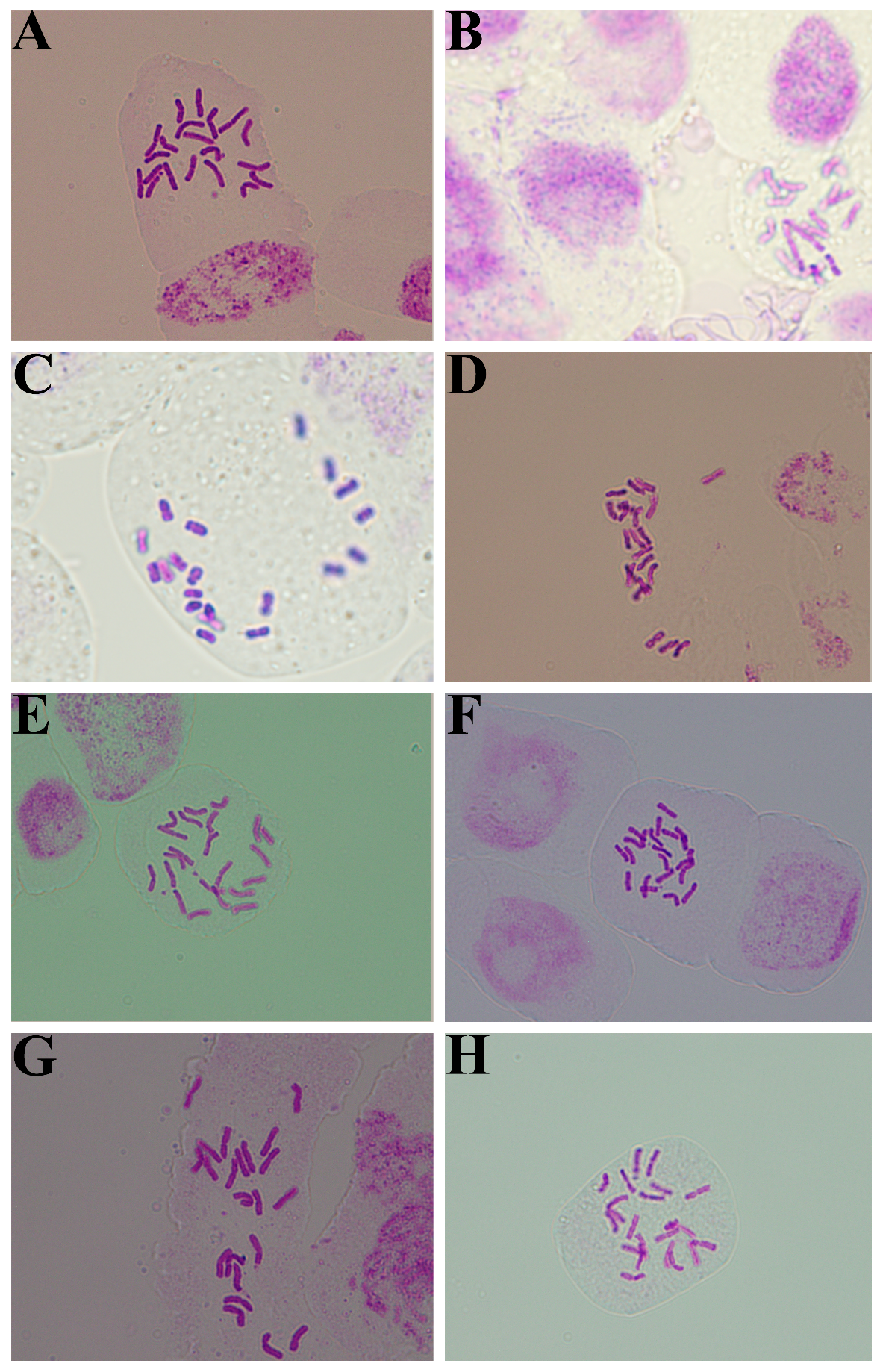


Figure S2. Chromosome numbers of *Peucedanum* s.l. plant. A: *P. dissolutum*; B: *P. mashanense*; C: *P. medicum*; D: *P. harry-smithii*; E: *P. harry-smithii* var. *subglabrum*; F: *P. huangshanense*; G: *P. praeruptorum*; H: *P. wawrae*.
